# Supplementary figures and images for: VPS35 and α-Synuclein fail to interact to modulate neurodegeneration in rodent models of Parkinson’s disease
Source: Mol Neurodegener. 2023 Aug 4;18:51. doi: 10.1186/s13024-023-00641-4 (PMC10403858; doi:10.1186/s13024-023-00641-4)

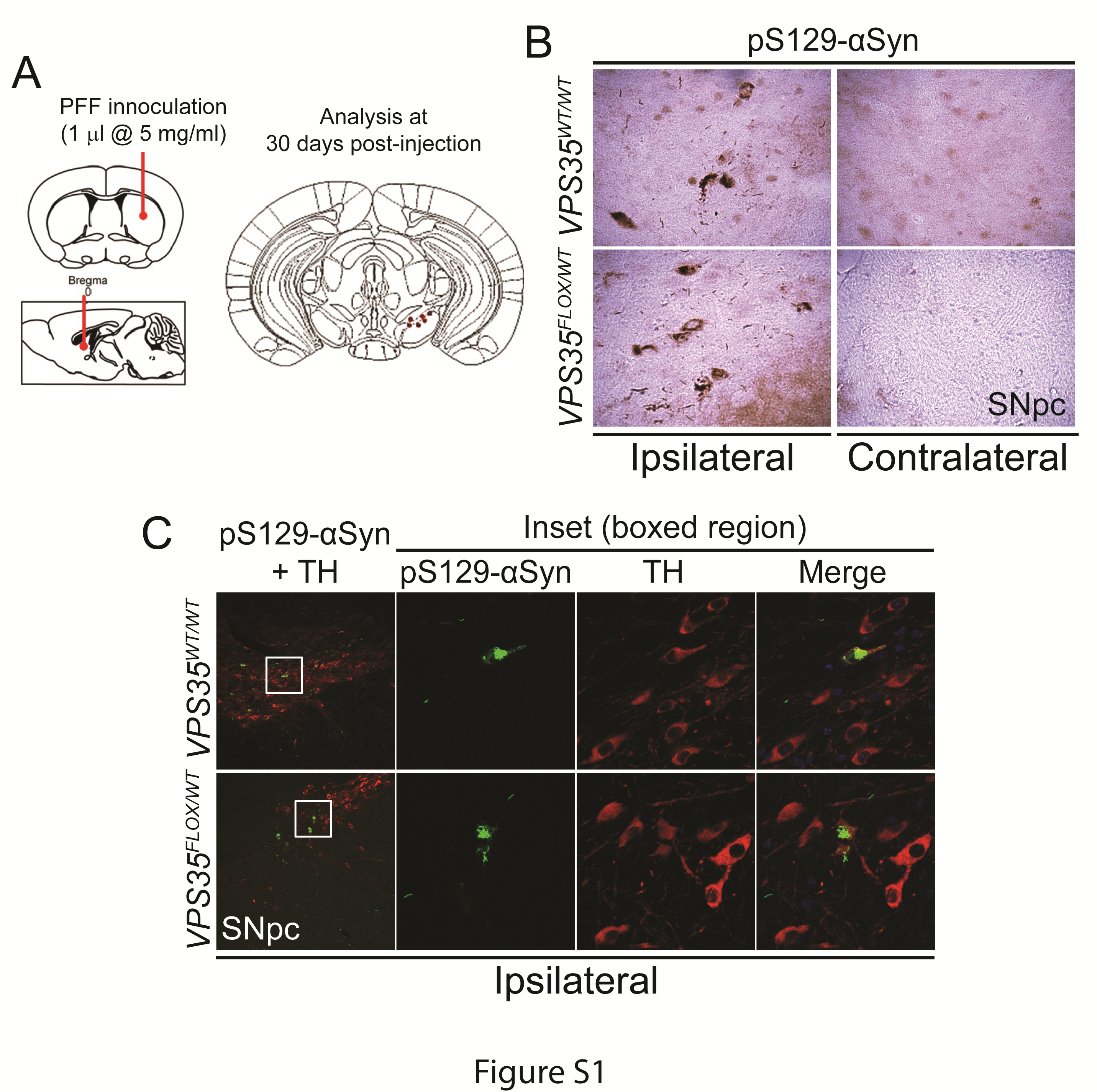

Supplement: Supplementary file 1 — Supplementary Material 1: Figure S1. Heterozygous VPS35 deletion does not alter the initial spread of α-Syn pathology in the α-Syn-PFF model. A) Schematic illustration of brain injection site. Top and bottom show coronal and sagittal planes, respectively. Red line indicates injection path and red dot indicates injection site. Mice were sacrificed 30 days after unilateral intrastriatal injection of 5 μg mouse α-Syn-PFFs. B) Representative images of pS129-α-Syn-positive immunoreactivity indicating equivalent α-Syn pathology/accumulation within the ipsilateral SNpc of age-matched VPS35WT/WT and VPS35FLOX/WT mice at 30 days post-injection. The contralateral SNpc lacks pS129-α-Syn-positive pathology, as expected. Scale bar: 100 μm. C) Immunofluorescent confocal co-localization of pS129-α-Syn-positive pathology in TH-positive dopaminergic neurons of the ipsilateral SNpc from VPS35WT/WT and VPS35FLOX/WT mice at 30 days post-injection of α-Syn-PFFs. pS129-α-Syn accumulates equivalently in the soma of dopaminergic neurons between genotypes. Data are representative of n = 3 mice/genotype. Scale bar: 15 μm [file 13024_2023_641_MOESM1_ESM.png]

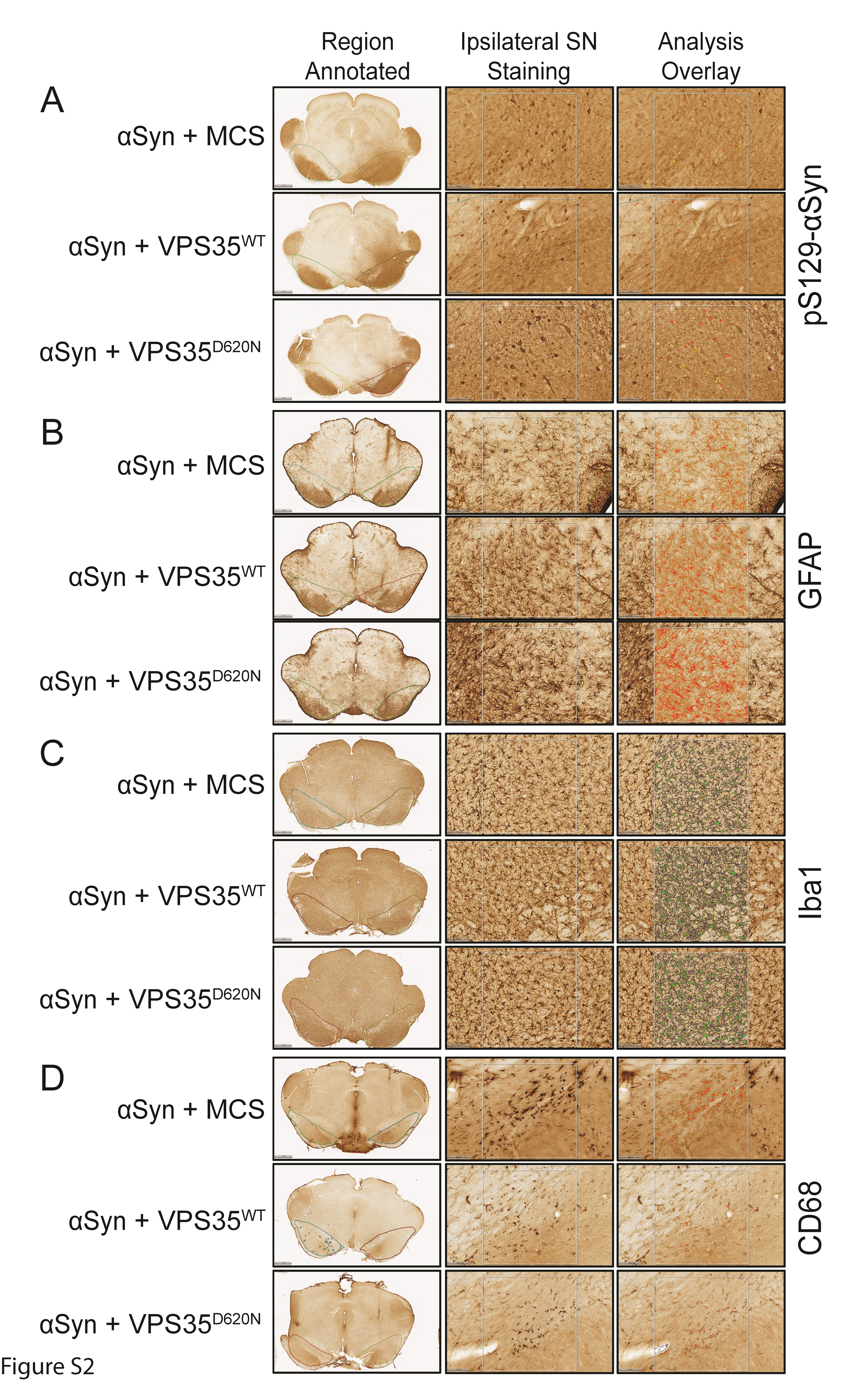

Supplement: Supplementary file 2 — Supplementary Material 2: Figure S2: Quantitative neuropathological analyses: Midbrain sections were digitized and tissue sections spanning the ipsilateral and contralateral substantia nigra (SN) were outlined manually in HALO software for automated quantitation. Once annotated, algorithms were applied that allowed detection of pathology using thresholding by optical density. Thresholds were individually optimized for each immunohistochemical stain to provide broad detection of pathology in brain regions with both high and low density pathology, without inclusion of background staining. Ipsilateral nigra regions are shown for (A) pS129-α-Syn, (B) GFAP, (C) Iba1, and (D) CD68 staining/pathology with or without an analysis overlay (red), which was used to quantify the percent area occupied by pathology/staining [file 13024_2023_641_MOESM2_ESM.png]

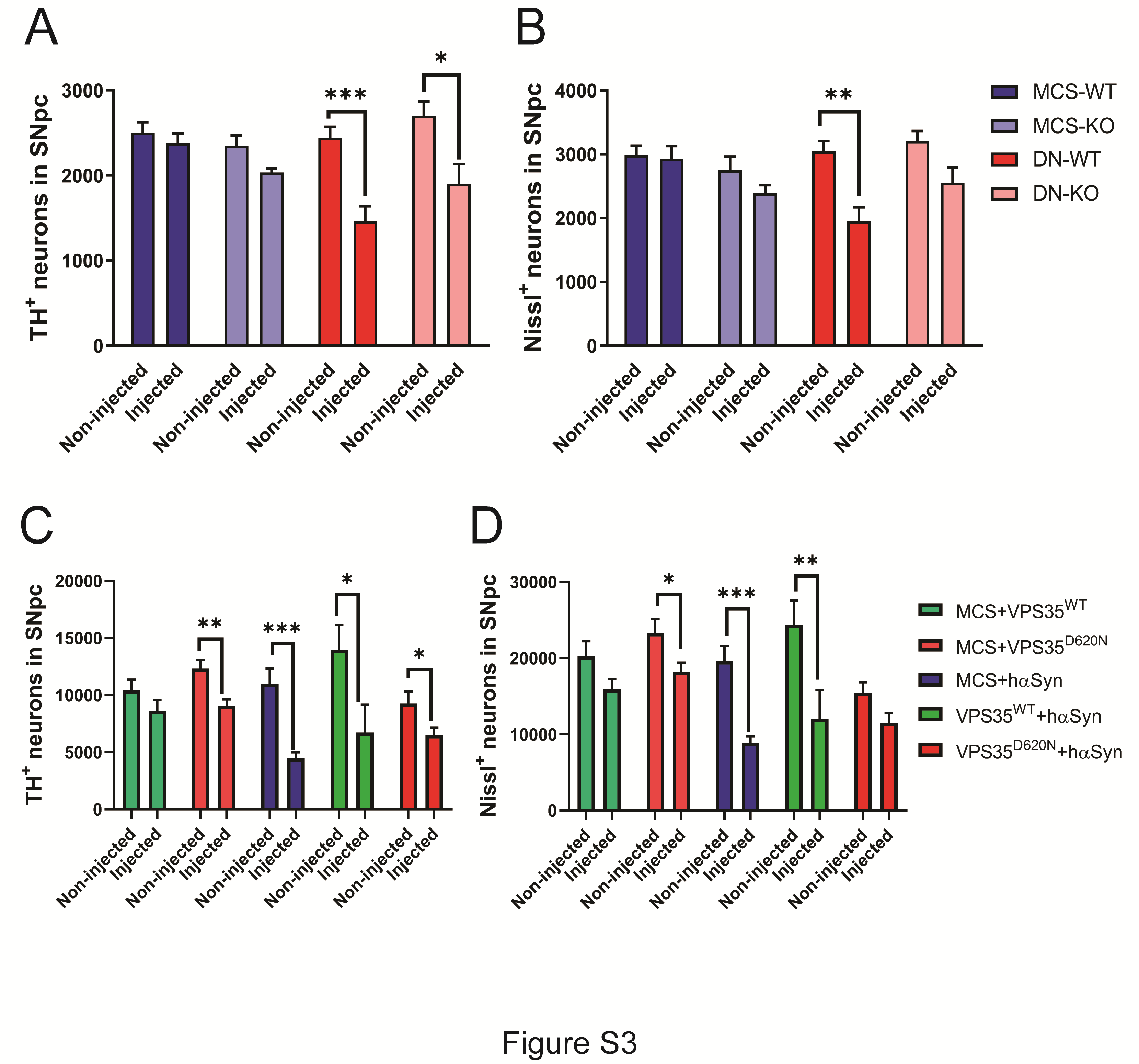

Supplement: Supplementary file 3 — Supplementary Material 3: Figure S3: Stereological cell counts. Stereological counts of the total number of TH-positive and Nissl-positive immunoreactive neurons in the SNpc of A-B)SNCA WT and KO mice induced by D620N VPS35 or control virus at 12 weeks (from Fig. 2C). Bars represent the mean ± SEM, n = 9–12 mice/group. *P < 0.05, **P < 0.01 or ***P < 0.001 two-tailed, unpaired Student’s t-test, as indicated. C-D) Stereological cell counts in the SNpc of AAV-αSyn/AAV-VPS35 co-injected rats at 14 weeks post-injection (from Fig. 6E). Bars represent the mean ± SEM, n = 7–8 rats/group. *P < 0.05, **P < 0.01 or ***P < 0.001 two-tailed, unpaired Student’s t-test, as indicated [file 13024_2023_641_MOESM3_ESM.png]

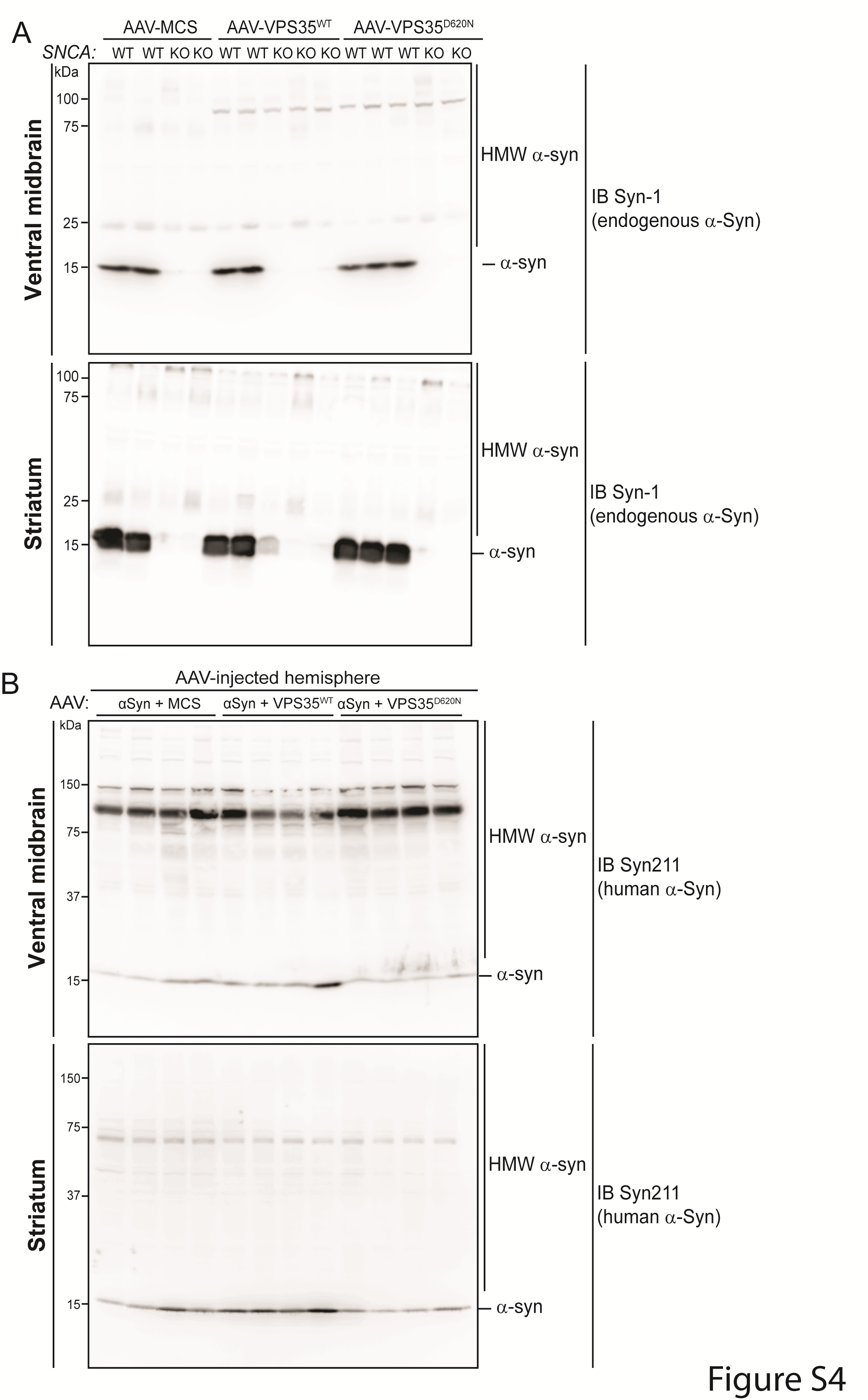

Supplement: Supplementary file 4 — Supplementary Material 4: Figure S4: Monomeric versus high molecular weight α-synuclein species: Full length Western blots that correspond to the representative blots of ventral midbrain and striatum extracts presented in main Fig. 3B (A) and 7 A (B). Blots were probed with antibodies to (A) total α-synuclein (Syn-1) or (B) human-specific α-synuclein (Syn211), respectively. Monomeric and high-molecular weight α-synuclein species are shown [file 13024_2023_641_MOESM4_ESM.png]
